# A Cautionary Tale of *Exophiala spinifera* Infection in Two Cats: Case Reports and Literature Review

**DOI:** 10.1155/crve/5572312

**Published:** 2026-01-07

**Authors:** Maryann D. Makosiej, Stephanie Anderson, Mandy A. Womble, Ashley Parsley, Hiroyuki Mochizuki, Petra Bizikova, Tim Chan, Ramón M. Almela, Laura M. Ribas, Lina C. Bilhalva, Andrea P. Santos, Fábio Brum-Rosa, Danielle Meritet, Francisco O. Conrado

**Affiliations:** ^1^ Department of Comparative Pathobiology, Cummings School of Veterinary Medicine, Tufts University, North Grafton, Massachusetts, USA, tufts.edu; ^2^ Department of Clinical Sciences, College of Veterinary Medicine, North Carolina State University, Raleigh, North Carolina, USA, ncsu.edu; ^3^ Department of Population Health and Pathobiology, College of Veterinary Medicine, North Carolina State University, Raleigh, North Carolina, USA, ncsu.edu; ^4^ Department of Clinical Sciences, Cummings School of Veterinary Medicine, Tufts University, North Grafton, Massachusetts, USA, tufts.edu; ^5^ Department of Veterinary Clinical Sciences, Purdue University College of Veterinary Medicine, West Lafayette, Indiana, USA, purdue.edu; ^6^ Purdue Institute for Cancer Research, West Lafayette, Indiana, USA; ^7^ Department of Comparative Pathobiology, Purdue University College of Veterinary Medicine, West Lafayette, Indiana, USA, purdue.edu

**Keywords:** chromoblastomycosis, feline medicine, fungal disease, muriform cells, opportunistic disease

## Abstract

This case series reports two independent cases of *Exophiala spinifera* infection in adult male neutered domestic cats, both referred following misdiagnosis. To date, only six cases associated with this organism have been reported in domestic cats, excluding those described herein. These also represent the first documented cases of *E. spinifera* infection in cats in the United States. In both cases, a definitive etiologic diagnosis could not be made by cytology, histology, or fungal culture. Moreover, histologic features did not allow for clear classification of the lesions as phaeohyphomycosis or chromoblastomycosis. Ultimately, accurate identification of the fungal pathogen was achieved through molecular diagnostic testing, rather than conventional mycologic or microscopic methods. These cases underscore the importance of molecular diagnostics and inter‐institutional collaboration in the accurate identification of dematiaceous fungi, such as *E. spinifera*, particularly given their variable clinical and pathological presentations.

## 1. Introduction

Dematiaceous fungi are a group of molds characterized by the presence of melanin or melanin‐like pigments in their cell walls [1]. This group includes various genera such as *Exophiala*, *Fonsecaea*, *Alternaria*, *Bipolaris*, *Cladophialophora*, and *Curvularia* [1]. Infections caused by these organisms are associated with various clinical presentations, including phaeohyphomycosis and chromoblastomycosis [2]. Histologically, phaeohyphomycosis is marked by the presence of yeast‐like cells, pseudohyphae, hyphae, or a combination of these structures, typically accompanied by a pyogranulomatous inflammatory infiltrate [3]. Chromoblastomycosis shares this inflammatory pattern but is defined by the presence of distinctive intermediate vegetative forms known as muriform cells [4]. Also referred to as sclerotic bodies or Medlar bodies, muriform cells are thick‐walled, round to polyhedral structures measuring 5–12 *μ*m in diameter, with multiple transverse septa [2, 4]. Although hyphae may occasionally be present, muriform cells represent the predominant fungal form observed in chromoblastomycosis [5].

In humans, chromoblastomycosis typically presents as verrucous, hyperplastic lesions, whereas phaeohyphomycosis exhibits a wider range of tissue involvement and gross morphological appearances [2, 3, 6]. In cats, the clinical presentation of dematiaceous fungal infections is less well‐defined but often includes cutaneous lesions such as dark nodules, plaques, or nonhealing wounds [7]. These lesions most commonly affect the pinnae, digits, or nasal planum [7]. In some cases, the infection may involve the upper or lower respiratory tract or disseminate systemically, particularly in immunocompromised cats [7].

*Exophiala spinifera* (family Herpotrichiellaceae) has a global distribution and ubiquitous presence in soil [8]. Infections caused by *E. spinifera* are rarely reported in cats; to date, only six cases have been documented, none of which occurred in the United States [9–13]. Here, we report two cases of *E. spinifera* infection in cats that could not be initially diagnosed using standard microbiological, cytological, or histological methods. These cases underscore the essential role of molecular diagnostics and interdisciplinary collaboration in accurately identifying uncommon fungal pathogens. They also highlight the difficulty of reliably distinguishing phaeohyphomycosis from chromoblastomycosis, given the overlapping clinical and histopathologic characteristics shared by these conditions.

## 2. Case Description

### 2.1. Case 1

A 2.5‐year‐old, neutered male, domestic long‐haired cat was presented to the North Carolina State University′s Dermatology Service for a recheck of a previously diagnosed feline atopic skin syndrome and assessment of a new, mass‐like lesion on the right forepaw. The patient had first been evaluated 1 year earlier for severe pruritus, self‐induced alopecia, and excoriations. Feline leukemia virus (FeLV) and feline immunodeficiency virus (FIV) were ruled out at that time (SNAP FIV/FeLV Combo Test, IDEXX Laboratories Inc., Westbrook, ME). During this first presentation, an elimination diet trial was initiated to exclude food allergy, and the cat was started on oral cyclosporine (6.9 mg/kg/day) and prednisolone (0.7 mg/kg/day). Due to only partial improvement in pruritus, prednisolone was replaced with triamcinolone (0.2 mg/kg/day) after 1 month. This regimen successfully managed clinical signs; however, repeated attempts to discontinue triamcinolone led to recurrence of pruritus and excoriations, necessitating continued treatment with cyclosporine (6.9 mg/kg/day) and triamcinolone three times weekly (0.2 mg/kg). Allergen‐specific IgE testing (ALLERCEPT Testing, HESKA, Loveland, CO) identified sensitivities to several grasses (*Sorghum halepense*, *Paspalum notatum*, and *Cynodon dactylon*), weeds (*Bassia scoparia*, *Salsola tragus*, *Rumex* spp., and *Plantago lanceolata*), and mites (*Dermatophagoides farinae*, *D. pteronyssinus*, *Tyrophagus* spp., *Acarus siro*, and *Blomia tropicalis*). Based on these results, subcutaneous allergen‐specific immunotherapy (ASIT) was initiated. At a 3‐month recheck, a 2.0 x 2.5 cm soft, ulcerated mass was noted between digits two and three of the right front paw (Figure 1a,b). According to the owner, the mass developed 12 months after starting cyclosporine and steroid therapy, and 1 month after initiating ASIT.

Figure 1Macroscopic appearance of lesions caused by *Exophiala spinifera* infection in two cats. Case 1 (a, b) and Case 2 (c, d). (a) Well‐demarcated, diffuse, erythematous, hyperkeratotic lesions. (b) Area of alopecia. (c) Multifocally ulcerated epidermis with diffuse swelling and crusting at initial presentation. (d) Erythematous, progressive ulceration of the paw pad epidermis at a 1‐month follow‐up posttreatment.

**Figure:**
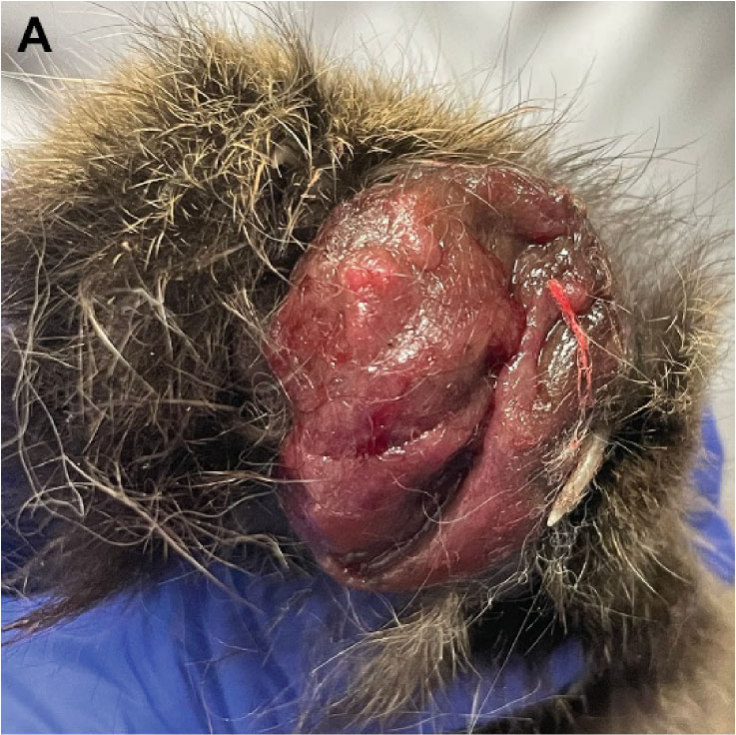
(a)

**Figure:**
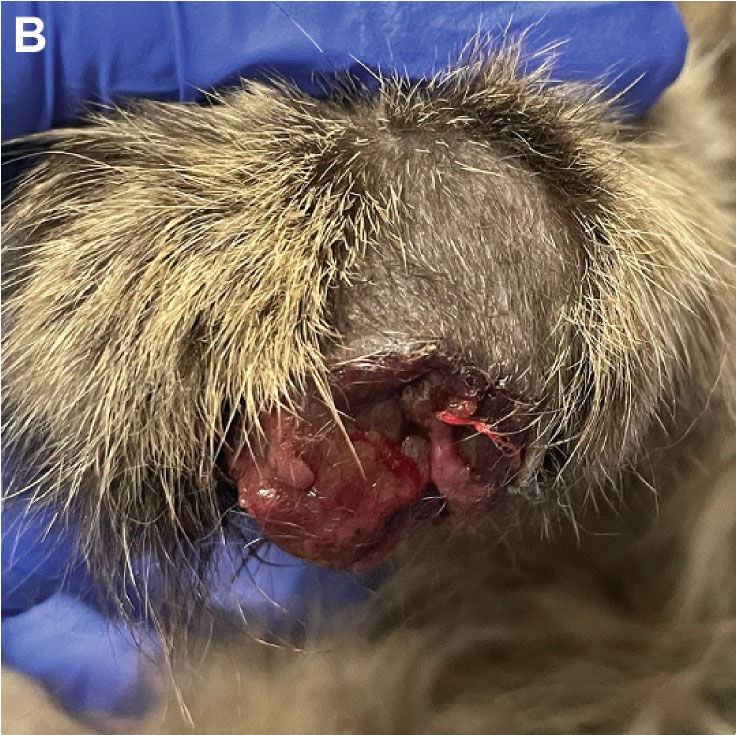
(b)

**Figure:**
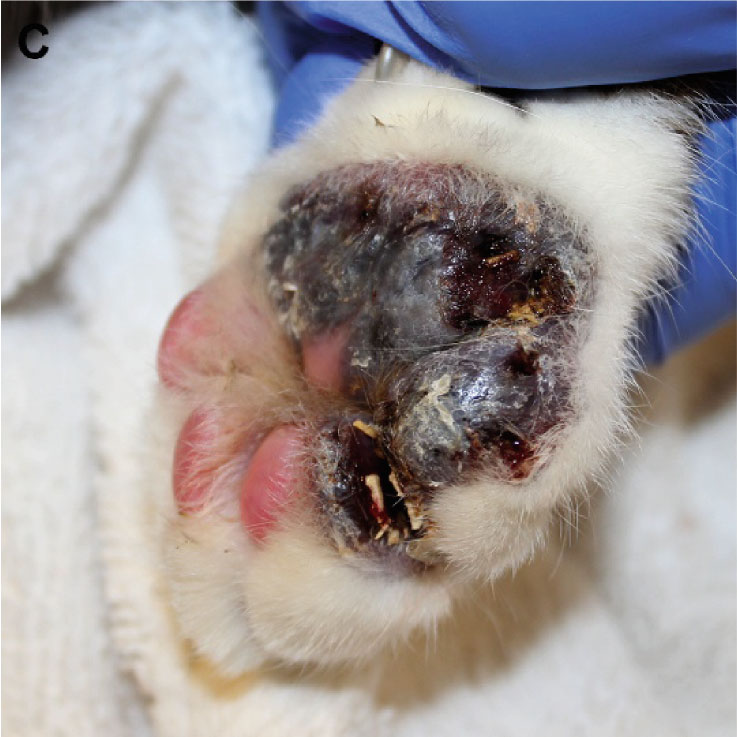
(c)

**Figure:**
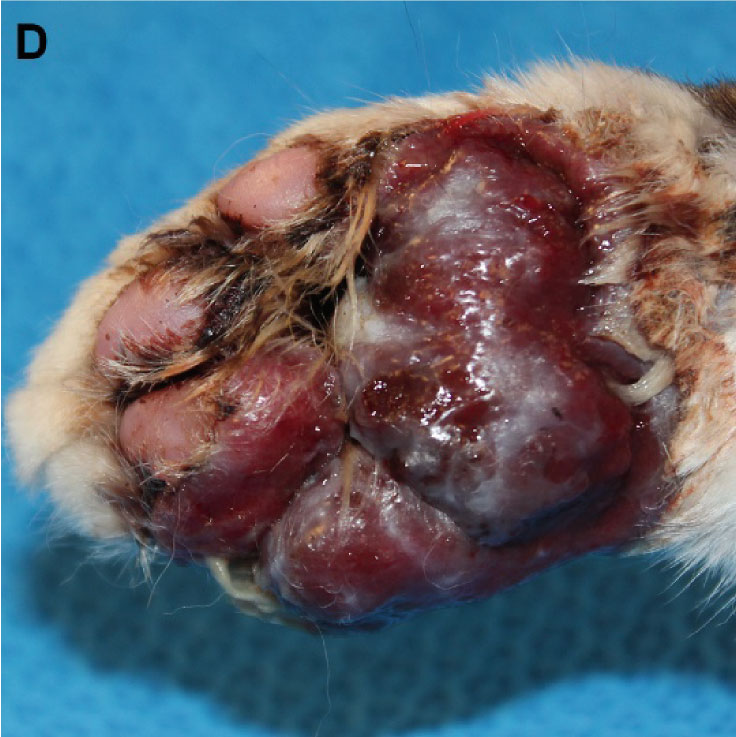
(d)

Given the appearance of the lesion, a fine‐needle aspirate was performed and submitted for cytologic evaluation. Preparations stained with Wright–Giemsa revealed a mixed inflammatory infiltrate composed of macrophages, multinucleated giant cells, neutrophils, and eosinophils. Scattered among the inflammatory cells were fungal organisms, appearing as round, blue–green, or pale/nonstaining structures measuring 5–15 *μ*m in diameter, often collapsing (Figure 2a,b). Occasionally, these structures were more basophilic with a thin, clear capsular border. No hyphae or pseudohyphae were observed. The cytologic interpretation was mixed inflammation with the presence of fungal organisms. Due to the atypical morphology, distinct from more commonly recognized fungal pathogens such as *Cryptococcus* and *Blastomyces* species, further diagnostics were pursued.

Figure 2Cytologic findings associated with *Exophiala spinifera* infection in two cats. Case 1 (a, b) and Case 2 (c, d). (a) Numerous fungal structures are present, both extracellular and phagocytized by macrophages. 50× objective. (b) Fungal organisms are seen within multinucleated giant cells. 100× objective. (c) Mixed inflammatory infiltrate composed of macrophages, nondegenerate neutrophils, lymphocytes, and plasma cells is seen. 50× objective. (d) Abundant intracellular and extracellular round fungal organisms are noted. 100× objective. Wright–Giemsa stain. Bar = 10 *μ*m.

**Figure:**
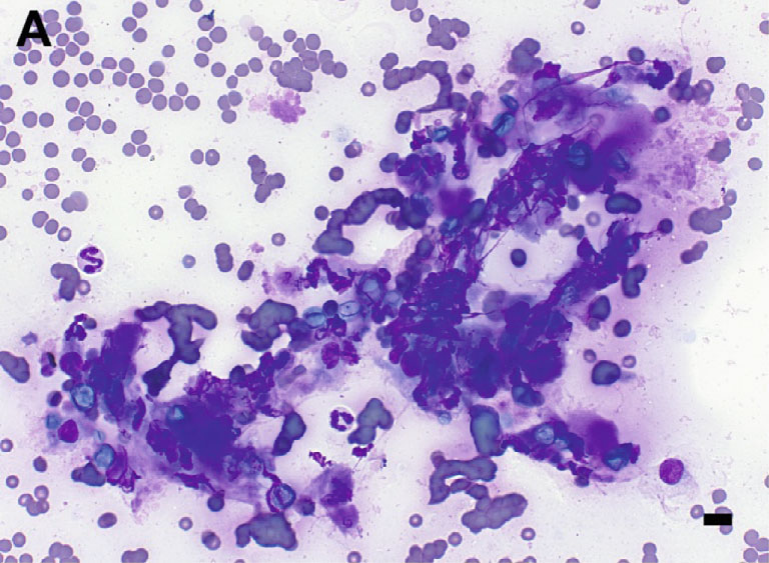
(a)

**Figure:**
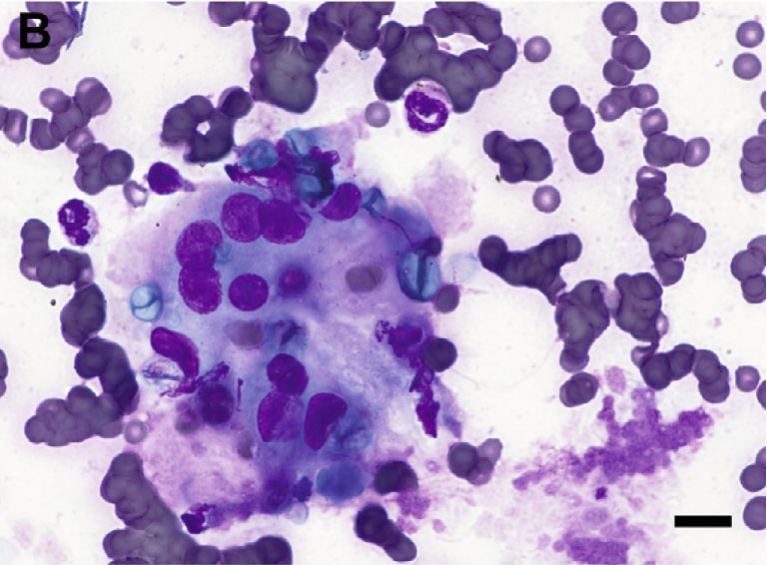
(b)

**Figure:**
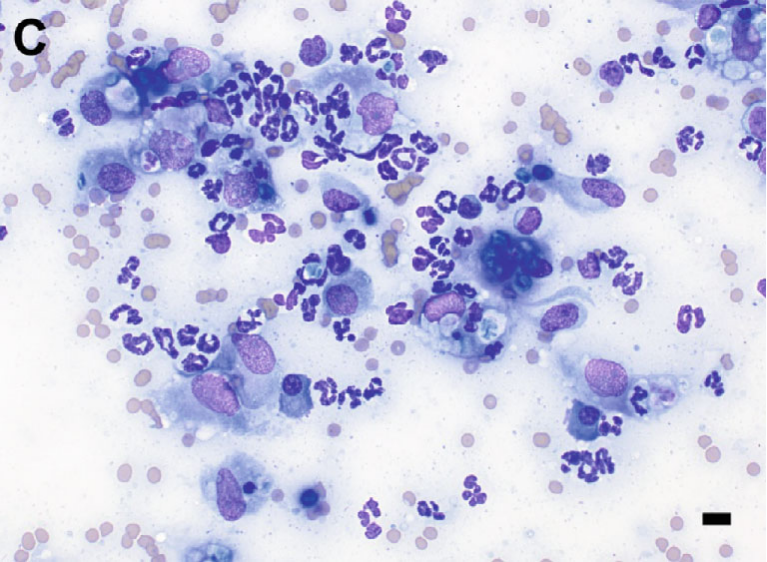
(c)

**Figure:**
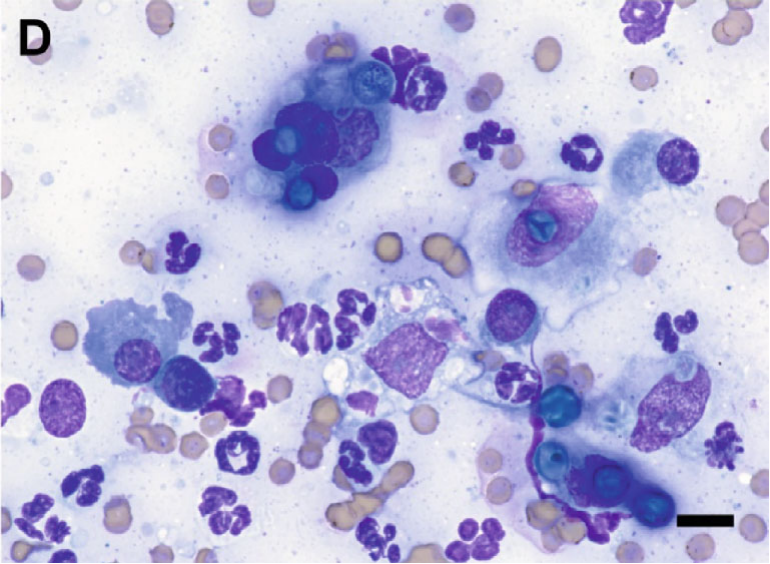
(d)

A biphasic fungal culture was performed using Columbia blood agar and Sabouraud dextrose agar supplemented with chloramphenicol. Duplicate plates were incubated at room temperature, 30°C, and 37°C for up to 4 weeks. Grossly, colonies appeared black to dark brown, with a downy texture and centrally raised tufts (Figure 3a). Microscopic examination revealed numerous apical, blue–green to pale, single‐celled ovoid conidia borne on septate conidiophores (Figure 3b). These characteristics led to a presumptive but equivocal identification of *Sporothrix schenckii*, as the isolate did not demonstrate clear yeast‐phase conversion at 37°C.

Figure 3Macro‐ and microscopic appearance of *E. spinifera* isolates from two cats. Case 1 (a, b) Case 2 (c, d). (a) The colony is black to dark brown, irregular, and centrally raised, with tufts of aerial mycelium. (b) Wet mount showing apical, blue–green to pale, ovoid conidia. (c) The colony is black to dark brown, mucoid, and yeast‐like. (d) Wet mount showing numerous budding, yeast‐like cells, some forming pseudohyphae.

**Figure:**
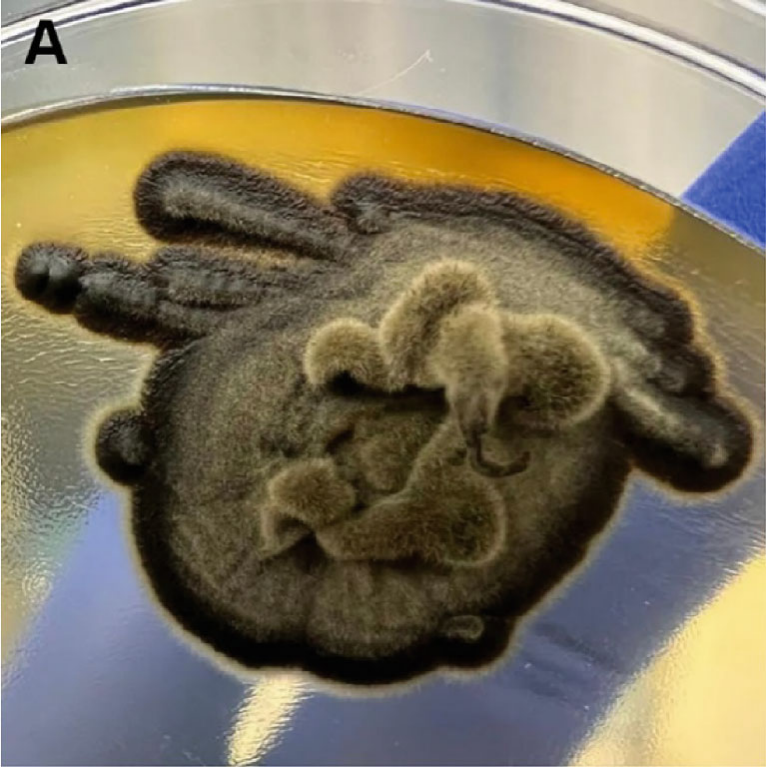
(a)

**Figure:**
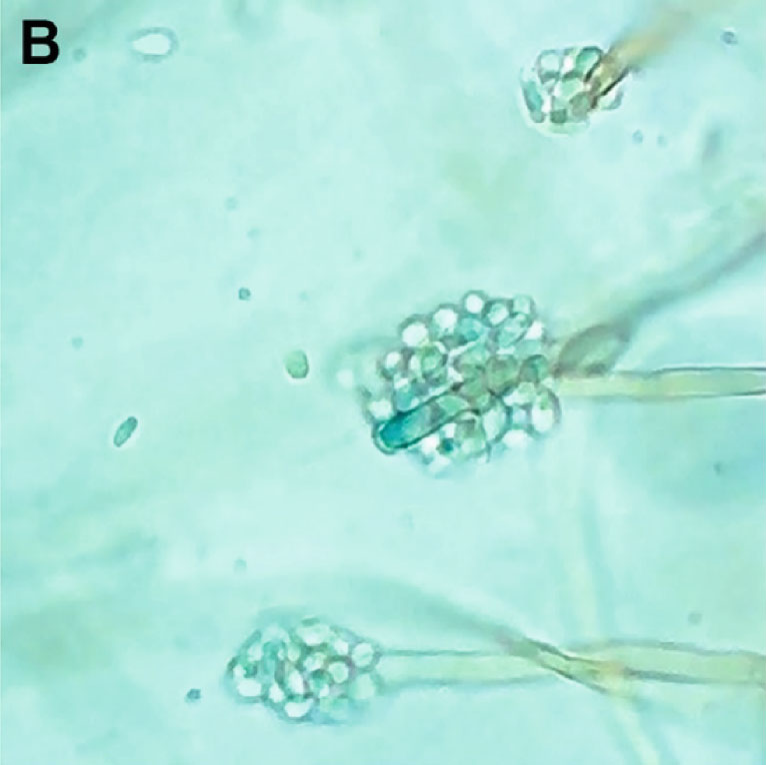
(b)

**Figure:**
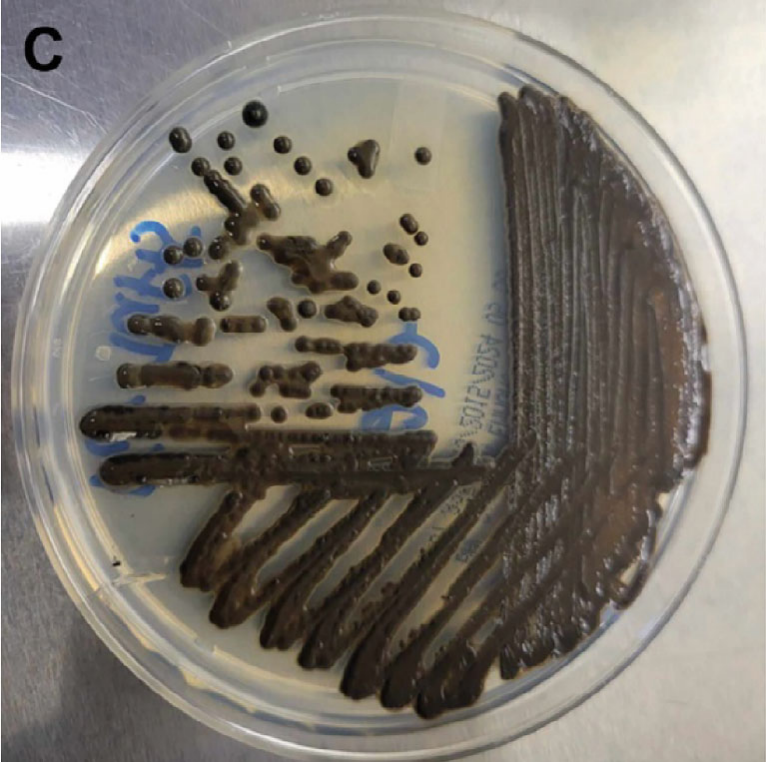
(c)

**Figure:**
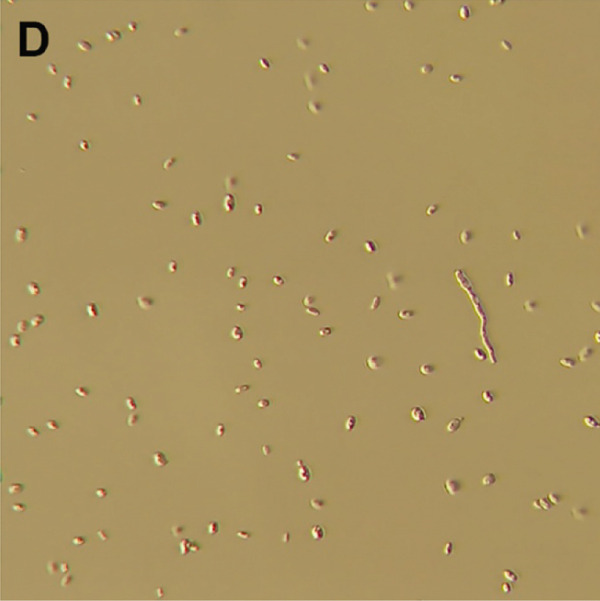
(d)

A surgical biopsy with histopathologic evaluation of the mass was also performed. Microscopic assessment revealed severe, ulcerative, granulomatous dermatitis with numerous intralesional fungal organisms (Figure 4a). These organisms were round, 5–12 *μ*m in diameter, with a light brown, refractile cell wall that was often folded, resembling the internal septation of muriform cells characteristic of chromoblastomycosis. Grocott′s methenamine silver (GMS) (Figure 4b) and periodic acid–Schiff (PAS) stains highlighted large numbers of pigmented, rounded fungal cells with rare evidence of budding.

Figure 4Photomicrographs of the histopathologic appearance of lesions caused by *E. spinifera* in two cats. Case 1 (a, b) and Case 2 (c, d). (a) The dermis is infiltrated by numerous epithelioid macrophages and multinucleated giant cells, mixed with intra‐ and extracellular pigmented, yeast‐like fungal organisms. H&E, 40× objective. (b) Pleomorphic, pigmented, yeast‐like organisms with rare budding, highlighted by GMS stain. 40× objective. (c) Numerous intrahistiocytic and extracellular pigmented, yeast‐like organisms associated with pyogranulomatous inflammation, effacing the dermis. H&E, 40× objective. (d) Yeast‐like organisms with variable budding (arrow), highlighted with PAS stain. 60× objective. Bar = 20 *μ*m.

**Figure:**
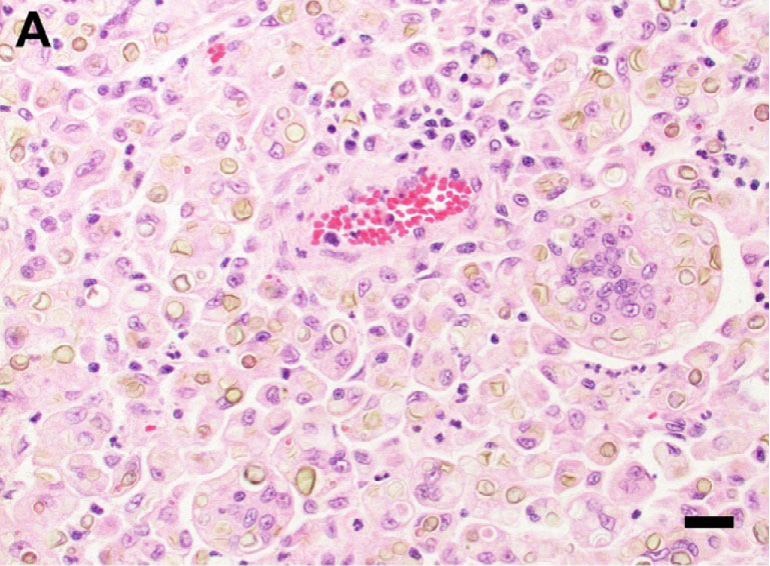
(a)

**Figure:**
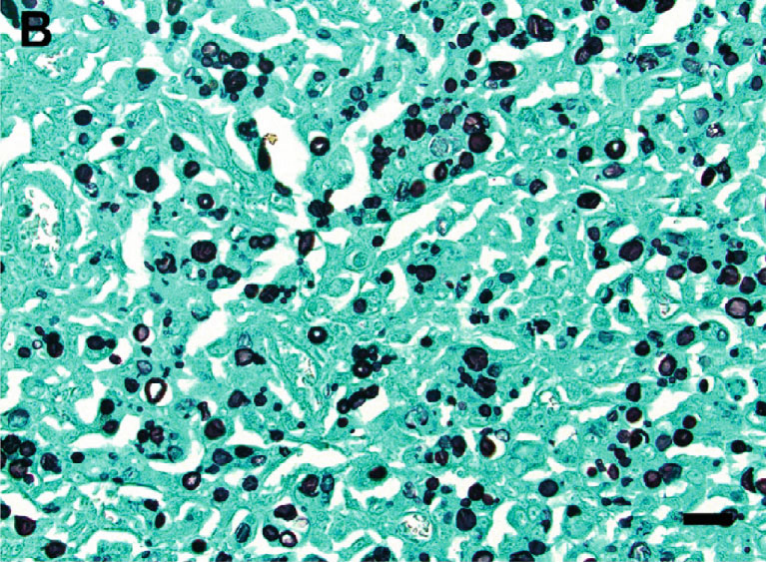
(b)

**Figure:**
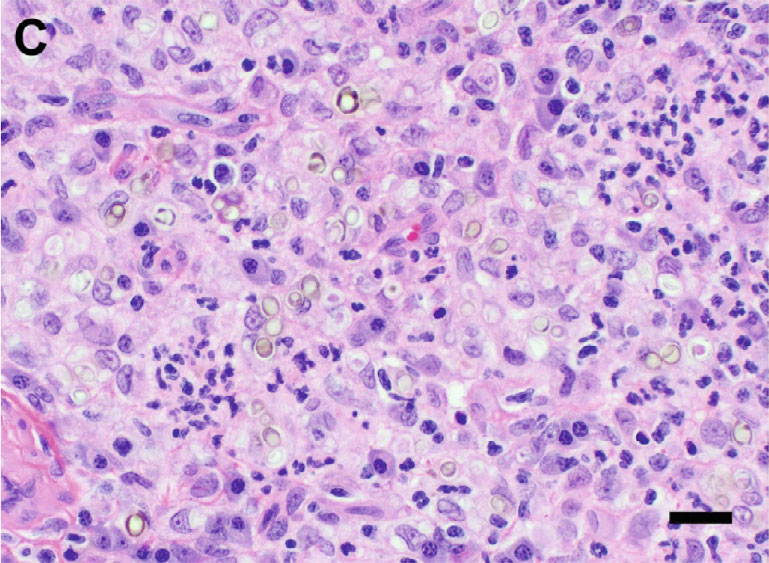
(c)

**Figure:**
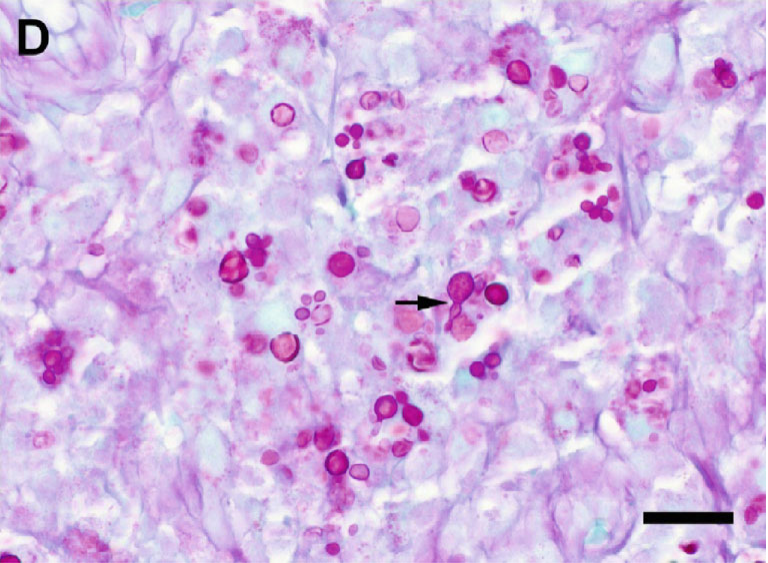
(d)

For species identification, molecular analyses were performed by the Veterinary Diagnostic Laboratory at the College of Veterinary Medicine, University of Illinois. Nucleic acids were extracted from scrolls obtained from formalin‐fixed, paraffin‐embedded tissue and amplified using primers targeting a portion of the 28S rRNA gene, as described previously [14]. Amplified DNA was purified sequenced using the Sanger method [15]. Following trimming of low‐quality bases and assembly, a 263 bp consensus sequence was submitted to the GenBank database [16] under Accession Number PQ699251. Comparison with the database showed 99.25% similarity to *E. spinifera* (GenBank entry: MH876941.1).

### 2.2. Case 2

A 5‐year‐old neutered male domestic medium‐haired cat was presented to the Dermatology Service at the Foster Hospital for Small Animals, Cummings School of Veterinary Medicine at Tufts University (TUCSVM), for evaluation of a nonhealing wound on the right forelimb paw (Figure 1c). The cat had been adopted as a kitten in Georgia and had since lived as an indoor‐only pet in New England, with supervised outdoor walks. The patient had previously tested negative for FeLV and FIV (SNAP FIV/FeLV Combo Test, IDEXX Laboratories Inc.). The lesion was first noted 1 year prior to this presentation, initially manifesting as two small wounds on the dorsal and ventral aspects of the right forelimb paw. At the initial visit to the primary care veterinarian, the cat received a single subcutaneous injection of cefovecin (8 mg/kg). Approximately 6 months later, a 5‐day course of marbofloxacin (2.3 mg/kg/day) and prednisolone (0.91 mg/kg/day) was prescribed, but no improvement was observed. Over time, the wounds progressed, expanding from the metacarpal pad to involve the fifth digital pad. At the time of the presentation to TUCSVM, physical examination revealed marked swelling, ulceration, hyperpigmentation, and multiple draining tracts with serosanguineous exudation affecting both pads.

Fine‐needle aspirates and impression smears of the lesion were performed for cytologic evaluation. Microscopic examination revealed numerous nondegenerate neutrophils, macrophages, lymphocytes, and plasma cells, as well as thin‐walled, round to oval fungal organisms (Figure 2c,d). These structures were small, blue–green, measuring approximately 5–10 *μ*m in diameter, and occasionally appeared collapsed or wrinkled. Macrophages occasionally exhibited an epithelioid appearance, often forming clusters and frequently containing phagocytized fungal cells. The cytomorphologic findings were consistent with mixed inflammation associated with fungal organisms but lacked features sufficient for species‐level identification.

For further characterization, fungal culture was performed at the Animal Health Diagnostic Center, Cornell University. Isolation was performed on Sabouraud dextrose Emmons and inhibitory mold agar, incubated at 30°C for 28 days. Macroscopically, a dark brown colony with a mucoid, yeast‐like appearance was observed (Figure 3c). A wet mount preparation revealed numerous yeast‐like budding cells, occasionally forming pseudohyphae (Figure 3d). Despite these findings, the atypical morphologic features precluded definitive species identification based on culture characteristics alone.

Despite an initial 26‐day course of itraconazole (1.8 mg/kg/day), followed by a 6‐week course of fluconazole (9 mg/kg/day), the skin lesions worsened (Figure 1d). The cat was subsequently treated with terbinafine (5.5 mg/kg/day) and itraconazole (9 mg/kg/day) for 1 week; however, due to worsening lesions and pain, a limb amputation was ultimately performed. The amputated limb was submitted for histopathologic evaluation, and terbinafine and itraconazole were continued postoperatively for 6 months. Microscopic examination revealed diffuse pyogranulomatous inflammation within the dermis, characterized by large numbers of neutrophils and epithelioid macrophages, with fewer lymphocytes and plasma cells. Numerous intrahistiocytic and extracellular pigmented fungal organisms, round to oval, measuring 5–15 *μ*m in diameter, with a double‐contoured cell wall, were observed (Figure 4c). Occasional budding of these fungal structures was also noted (Figure 4d).

Polymerase chain reaction was performed at the Clinical and Molecular Pathology Research Laboratory, Purdue University, to identify the organism. Fresh‐frozen tissue and pure colonies from the fungal culture were used for DNA extraction [17], and amplification was performed using a primer pair targeting the ITS2 region [18]. Amplicons were purified and sequenced using the Sanger method [15]. After trimming and assembly, an identical 341 bp consensus sequence was obtained from both samples and submitted to GenBank [16] under Accession Number PQ699719. Database comparison revealed 100% similarity to *E. spinifera* (GenBank entry: OQ466703.1).

Six months after limb amputation, while continuing antifungal therapy, the patient was presented for evaluation of recurrent sneezing and upper respiratory congestion. An endoscopic biopsy of the nasal mucosa was performed for histopathologic analysis. The lesion was characterized as pyogranulomatous rhinitis with intralesional pigmented fungal organisms, presumed to be the same species identified in the paw lesion. The dose of itraconazole was increased to 18 mg/kg/day, while the same dose of terbinafine was continued, with no apparent clinicopathologic adverse effects. The patient′s fungal rhinosinusitis demonstrated partial improvement with the adjusted treatment, and case management is ongoing at the time of writing.

## 3. Discussion

This study demonstrates the challenges of diagnosing *E. spinifera* using traditional methods such as cytology, histology, and fungal culture. These challenges are largely attributable to the organism′s variable morphological features, which often overlap with those of other fungi [19, 20]. Consequently, in our cases, as in others, accurate species identification frequently required molecular diagnostic techniques [5, 20].

Consistent with our findings, prior studies in both humans and cats have shown that species‐level identification of black‐pigmented fungi is rarely achievable through histologic evaluation alone [21, 22]. This limitation arises because various dematiaceous fungi can produce similar histologic patterns [6]. Additionally, the cytomorphology of *E. spinifera* is highly variable, likely reflecting the poorly understood clinical spectrum of phaeohyphomycosis and chromoblastomycosis [5]. Although some reports describe these organisms as spherical elements [12], others report a predominance of yeast‐like cells and long, septate hyphae [9, 23]. In the present cases, cytologic evaluation revealed primarily rounded forms of *E. spinifera*, which can resemble true yeast. As a result, more common yeast infections such as blastomycosis and cryptococcosis were initially considered; however, these were ultimately ruled out based on inconsistent cytomorphologic features, including differences in size and the absence of a distinct, thick capsule.

Fungal culture, although often employed to aid identification, is not always definitive. Identification of *E. spinifera* in culture requires prolonged incubation and interpretation by experienced specialists [6]. Moreover, microscopic features are often highly similar across species within the genus [6, 8, 21]. Fungal morphology in culture may also be influenced by factors such as the organism′s life stage, the composition of the growth medium, environmental conditions, nutrient availability, pH, temperature, and prior antifungal therapy [24, 25]. Although mycologic examination successfully identified *E. spinifera* in three previously reported feline cases [9–11], the biphasic culture in our series did not yield a species‐level diagnosis in Case 2 and resulted in an equivocal presumptive identification in Case 1. When grown at 25°C, *S. schenckii* produces hyaline, septate hyphae, and conidial groups that may resemble the ovoid conidiophores and ellipsoidal conidia sometimes seen in *E. spinifera* [8, 9, 26]. However, a true yeast‐phase conversion at 37°C (expected for thermally dimorphic *Sporothrix* spp.) was not observed, and the characteristic cytologic features of sporotrichosis (including pleomorphic, oval to fusiform, or round yeast forms measuring 1–5 *μ*m by 2–10 *μ*m with a thin clear halo) [26] were absent. In fact, fungal culture results were incongruent with cytologic findings, further complicating the diagnostic process. It should also be noted that antifungal susceptibility testing was not performed for either isolate, although it may have provided valuable guidance for targeted therapy.

In the few previously reported cases of *E. spinifera* infection in cats, only three employed molecular diagnostics for species confirmation, with lesions presenting as multifocal cutaneous involvement affecting the paws and nasal bridge [9, 12, 13]. Among the remaining reports, one described a focal cutaneous lesion of the paw [11], and two others documented involvement of the nasal cavity [10, 11]. Given its environmental ubiquity, *Exophiala* spp. infections are commonly associated with traumatic inoculation [7, 9, 19]. This presumed route of transmission is supported by the frequent involvement of the paws in affected cats, including the cases described herein. Although such infections can occur in otherwise healthy individuals, immunocompromised cats may be predisposed to more severe disease [7]. In Case 1, the temporal association between immunosuppressive therapy (cyclosporine and corticosteroids) and the onset of clinical signs raises suspicion that immunomodulation may have contributed to disease development.

These cases also illustrate the difficulty of distinguishing between phaeohyphomycosis and chromoblastomycosis, a distinction that requires integrated assessment of clinical presentation, histopathologic findings, and fungal morphology [4]. Even in human medicine, where these infections are better characterized, clinical and microscopic findings do not always align [27]. This discrepancy is understandable, as these conditions represent a spectrum of disease [27]. In veterinary medicine, classification criteria remain poorly defined and are the subject of ongoing debate [28]. For example, the only reported case of chromoblastomycosis in a cat, caused by *Cladophialophora carrionii*, was characterized solely by the presence of hyphae [29]. Conversely, in a case of feline phaeohyphomycosis caused by *E. spinifera*, the authors observed only round structures resembling muriform cells, with no hyphae identified [12]. In the cases described herein, the majority of fungal structures morphologically resembled muriform cells, which might suggest chromoblastomycosis. However, despite the use of routine histologic evaluation and special stains, inconsistencies in the literature precluded definitive classification.

This case series cautions against relying solely on cytologic, histologic, or culture‐based morphology for the diagnosis of fungal infections and underscores the importance of molecular testing for accurate species identification. A definitive diagnosis is best achieved through a combination of complementary diagnostic techniques, with molecular analysis serving as a critical component. In addition, these cases highlight the value of interdisciplinary collaboration. Through shared image review and multidisciplinary discussion, diagnosticians were able to recognize inconsistencies that prompted further testing, ultimately leading to accurate diagnosis and appropriate case management.

## Conflicts of Interest

The authors declare no conflicts of interest.

## Author Contributions

Maryann D. Makosiej and Stephanie Anderson contributed equally to this work. Danielle Meritet and Francisco O. Conrado are co‐senior authors.

## Funding

No funding was received for this manuscript.

## Data Availability

The data that support the findings of this study are openly available in GenBank at https://www.ncbi.nlm.nih.gov/genbank/ (Reference Number PQ699251; PQ699719).
